# A Proposition for the Estimation of the Maximum Tensile Strength of Variously Charged Nanocellulosic Film Materials Provided by Vacuum Filtration

**DOI:** 10.3390/nano11020543

**Published:** 2021-02-20

**Authors:** Tom Lindström

**Affiliations:** 1 Royal Institute of Technology, 100 44 Stockholm, Sweden; toml@kth.se; 2 StonyBrook University, Stony Brook, NY 11733, USA

**Keywords:** nanocellulose, tensile strength, delamination, charge density

## Abstract

This short investigation deals with a review of the tensile strength properties of six different types of nanocellulose films (carboxymethylated, carboxymethylcellulose-grafted, enzymatically pretreated, phosphorylated, sulfoethylated, and alkoxylated nanocellulose films) manufactured using identical protocols and the determination of the apparent nanocellulose yield of the same nanocelluloses and their tensile strength properties at different extents of delamination (microfluidization). The purpose was to test a previously suggested procedure to estimate the maximum tensile strength on these different procedures. A second goal was to investigate the impact of the nanocellulose yield on the tensile strength properties. The investigations were limited to the nanocellulose research activities at RISE in Stockholm, because these investigations were made with identical experimental laboratory protocols. The importance of such protocols is also stressed. This review shows that the suggested procedure to estimate the maximum tensile strength is a viable proposition, albeit not scientifically proven. Secondly, there is a relationship between the nanocellulose yield and tensile strength properties, although there may not be a linear relationship between the two measures.

## 1. Introduction

During the past decades, there has been an immense interest in nanocellulose materials, because of the possibility of substituting fossil materials with biodegradable materials and high strength nanocellulose materials produced from a multitude of sustainable resources. Nanocellulosic materials encompass at least three large type of materials: bacterial nanocellulose (BNC), nanocrystalline cellulose (CNC), and nanofibrillated cellulose (CNF), which can be produced with various functional groups and used for many different purposes. There are numerous reviews in the field, e.g, [1,2,3,4,5,6,7] and their various applications have also been highlighted, e.g., [8,9,10].

The strength properties of CNF films have previously been discussed in detail [11] in terms of effects like degree of polymerization, porosity, moisture content, etc., to which the reader may be referred to. There are also some important recent papers discussing ways to improve the strength properties of nanocellulose films that may give the reader an interesting perspective [12,13,14].

Fibers are in general stronger than their bulk properties due to surface defects, internal stresses, etc., during the manufacture of their bulk materials. The smaller the material entities, the less the possibility to find defects, but very little is known about structural defects in nanocellulose material, and there is little knowledge about the maximum strength of nanocellulosic film materials.

Some years ago, our laboratory was reinvestigating the well-known Page equation [15] in a papermaking exercise [16] using various dry strength additives. Interestingly, it was found that the Page equation could be proven from first principles. In addition, it was also found that when the BET-area of the investigated papers was plotted versus the tensile index, there was a straight line. As we knew fairly early that CNF-films with high oxygen barrier properties were non-porous [17] we could extrapolate the tensile index to a BET-area to zero m^2^/g. When it was found that the deduced tensile index was very close to the values determined by some reported strength properties of CNF-films, it was suggested that the line crossing the abscissa would be the maximum tensile strength of the CNF-film made from a certain pulp. This suggestion was discussed in some detail in a separate publication [18].

One problem with a more in depth investigation of this idea revealed, however, that there is a lack of universally accepted protocols for sample preparation and analysis of CNF-based films, though it has been suggested that standards for plastics and composites, such as ASTM D 638-01, ASTM D3039/D3039M-14, and ISO 527-1 have been suggested for CNF materials [19].

An investigation of the tensile protocols for CNF-testing also reveals a broad range of protocols, see Table 1.

**Figure 1:**
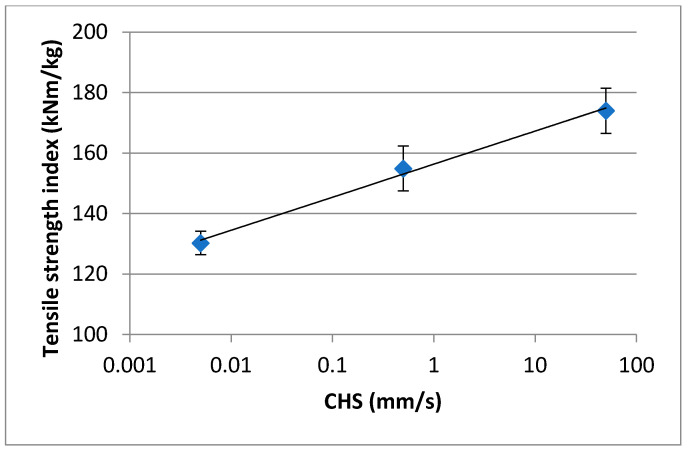
The effect of cross-head speed on the tensile strength of a nanocellulose film.

As an example, Figure 1 shows that the crosshead speed has a very significant impact on the measured properties for a nanocellulose film.

Another problem is that it is very difficult to determine the density of nanocellulose films, because the grammage must be low in order to minimize internal stresses induced by drying when a large amount of water needs to be replaced. Common thickness measurements for the density of CNF-films are difficult if the CNF film is not perfectly flat, as the modulus of films are very high, as we have discussed in an early paper [17].

Secondly, the film must be perfectly horizontal in order not to yield differences in basis weight in different parts of the film. It has also been realized that our papers prior to the [17] paper were not perfect in monitoring the density. The use of white-light interferometric profilometers and density gradient columns are useful for the purpose.

In this investigation, we have therefore reviewed our data from previous published investigations as we used the same original pulp, the same protocols, and the same type of delamination equipment (microfluidizer), in order to scrutinize the properties of six different nanocellulose films with respect to the idea that the maximum strength could be estimated from a BET-area tensile strength graph, as suggested in the [18] paper.

It was also of interest to investigate the possible relationship between the apparent nano-yield during delamination and the evolution of the tensile strength properties in this process.

## 2. Material and Methods

### 2.1. Materials

All nanocellulose materials were made from a never-dried commercial bleached sulphate dissolving pulp (trade name Dissolving Plus) from a mixture of Scotch Pine (40%) and Norway Spruce (60%) produced at Domsjö Fabriker (Aditva Birla Group, Örnsköldsvik, Sweden. The hemicellulose content of the pulp was 4.5% (*w/w*) (measured as solubility in 18% NaOH, R18) with a lignin content of 0.6%.

### 2.2. Methods

The protocols are all given in the cited papers, but as a courtesy to the readers, the most important features of the protocols are briefly given below:

### 2.3. Determination of Nano-Yield in CNF Materials

A gravimetric method using centrifugation was used to estimate the nano-yield in the CNF systems. In short, 0.02% (*w/w*) samples were prepared by first blending overnight (using a magnetic stirrer for approximately 18 h at 100 rpm concentrated CNF systems (2% (*w/w*)) in deionized water. These samples were thereafter centrifuged at 1000× *g* for 15 min. to remove larger constituents (e.g., residual fibre fragments). In a previous communication [20] the apparent overlap concentration of carboxymethylated CNF was found to be in the range of 0.04–0.07%, and below this concentration, larger constituents could be readily removed by centrifugation. Above the overlap concentration, all fibrils are removed from the suspension, and hence, this is a prerequisite to determine the apparent nano-yield fraction.

### 2.4. Film Preparation Procedure

CNF-suspensions with a dry content around 0.1% (*w/w*) were prepared by blending an appropriated amount of 2% CNF microfluidized materials with deionized water in a magnetic stirrer for around 18 h at 1000 rpm. The suspension was then degassed for one hour. Films (grammage: 30 ± 3 g/m^2^) were prepared through vacuum filtration using a 0.65-micron DVPP filter (Millipore) followed by drying under restrained conditions in an oven for 7 h at 50 °C.

### 2.5. Tensile Properties

Tensile strength properties were measured using an MTS tensile strength machine with a Teststar IIS controller (MTS Systems Norden AB, Askim, Sweden). The samples were equilibrated at 50% RH/23 °C for at least three days before conducting the measurements. The samples were weighted after the strips were cut out. The length and the width of the strips were 45 and 6 mm, respectively, and the distance between the grips holding the strips was 30 mm. The strips were then mounted into a tensile strength machine, and the mechanical properties were measured with a speed of 100%/min.

## 3. Results and Discussion

**Figure 2:**
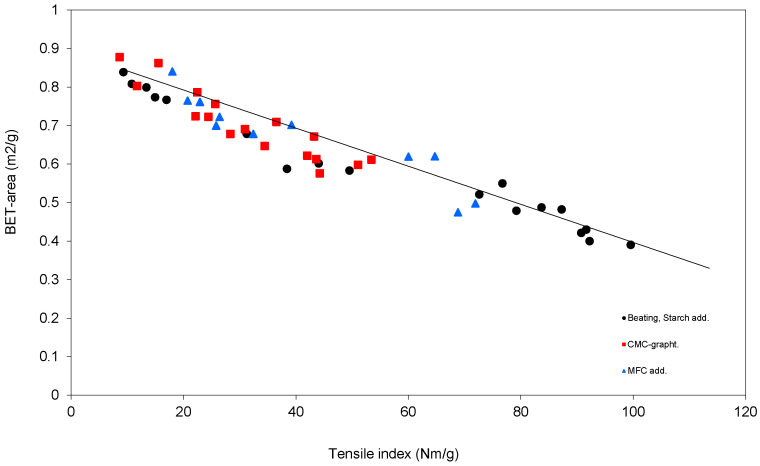
The relationship of the BET-area and the tensile index of nanocellulose films made from bleached paper pulp, when changing various dry strength agents (reproduced from Ref. [16] with permission from Royal Institute of Technology, Stockholm).

The idea that the ultimate strength of nanocellulose films could be approximated by using the extrapolation of the graph is shown in Figure 2, showing the strength of a paper in the presence of various dry strength additives and beating. If the straight line is extrapolated to where the line crosses the abscissa (172 Nm/g), it was suggested [18] that this shows the maximum strength value of the nanocellulose provided it has no porosity. Interestingly, the short-span strength of a paper made from the used pulp was 174 Nm/g, which is not surprising, as the pulp has a very low fibril angle if nanocellulose films do not show defects or internal stress defects and was suggested to be the maximum strength of the films.

Another feature of Figure 2 is that there is more or less a straight line, which may be puzzling, because the strength additives (also including beating) were all different, albeit the fact that they were all carbohydrates. Other dry strength agents, such as various synthetic polymers, would most likely not have resulted in the straight line in Figure 2.

In order to scrutinize this idea, some of our previous papers were investigated. The selection of these papers was based on the same type of pulp to make the CNF materials and the same type of delamination (microfluidizer), and finally, publications where the content of the nanocellulose on the delaminated fibres were investigated, see Table 2. As investigated by several authors, e.g., [21] the counterion size and valency is critical and must be a fixed protocol. In these investigations, the protocol was accordingly transferred to their COONa-form.

**Figure 3:**
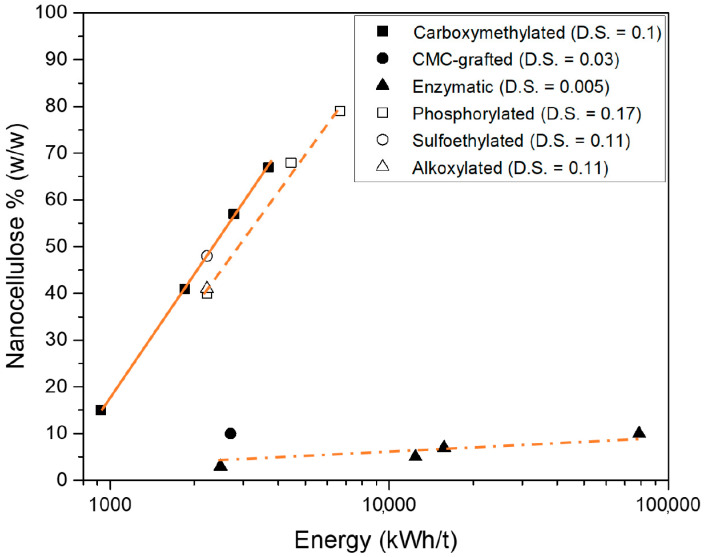
The evolution of the nanocellulose content in various types of CNF-materials (references are given in Table 2).

It should be noted that the very high energy levels are due to the low solid concentrations during delamination. In commercial practice, higher concentration levels are used, which significantly decreases the energy levels. For highly charged materials, the electrostatic repulsion between the fibrils also prevent clogging due to flocculation [27], and for enzymatically treated materials, there is lower viscosity (because of less delamination, see Figure 3), and the delamination can be operated at higher consistencies [22] Secondly, microfluidizers are a laboratory gadget. In commercial operations, high-pressure homogenizers are used, and these are not as sensitive to clogging compared to microfluidizers and can also be used at higher solid concentrations.

Figure 3 shows the effect of the nano-yield for the six different nanocellulose generations vs energy input for the delamination investigated in this context. The procedure for centrifugation has been discussed in previous papers by Naderi et al. [7,20] and is briefly described above in the method section. The key issue is that centrifugation must be performed below the critical overlap concentration in order to determine the nanocellulose content.

Considering the effects of charge density on the nano-yield, it is obvious that the higher the charge density, the higher the nano-yield. A similar order was found by Iwamoto et al for TEMPO-oxidized CNF materials [28]. The enzymatic CNF has an insignificant nano-yield. Hence, there is a similar order in nano-yield irrespective of the charged group in this sequence.

**Figure 4:**
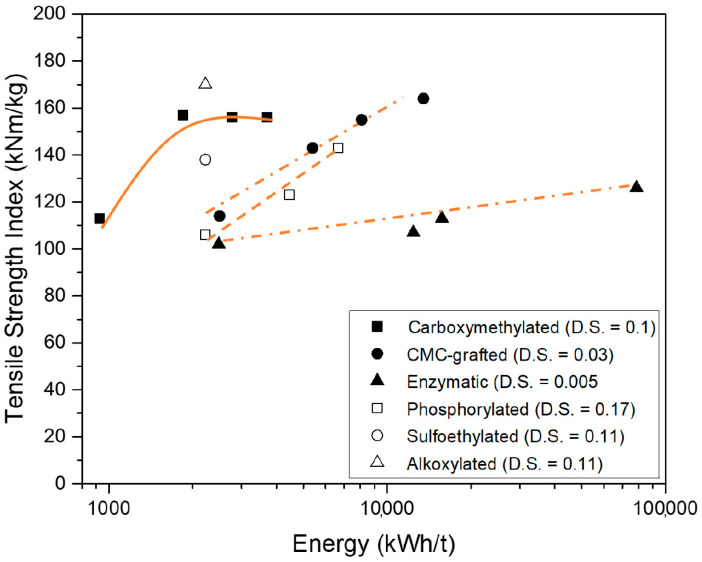
The evolution of the tensile index vs the energy input (references in Table 2).

Figure 4 shows the effects on the tensile index for the different CNF materials. It is known from our investigations that carboxymethylated CNF has a saturation (maximum) tensile strength up to a nano-yield around 70% [29], and it is also known that at this point, the carboxymethylated CNF is basically non-porous (Aulin et al. 2010). Unfortunately, there is a lack of densities for the other CNF-materials in Table 2. From Table 2, it can be concluded that the tensile strength values are slightly lower than 172 Nm/g for the carboxymethylated pulp (166 Nm/g), and the CMC-grafted has a tensile strength of 150 Nm/g in one investigation (the nano-yield is taken from this investigation), but in a later investigation of the CMC-grafted CNF, the tensile strength peaked at 164 Nm/g. The alkoxylated CNF had a tensile strength of 170 Nm/g, all approximately in line the estimations from Figure 2. The phosphorylated CNF and the sulfoethylated had a tensile strength index slightly lower, which may be explained by insufficient delamination, and has not yet reached its levelling-off level with respect to the tensile strength, and has most likely not reached the non-porosity level [29].

The low tensile strength of the enzymatic treated pulp is not an aberration, but it is conceived that the delamination has not reached its levelling-off point with respect to the tensile strength. All charged nanocellulose films have a high colloidal stability because of a sufficiently high charge density. The enzymatically treated nanocellulose, however, is inherently unstable, but high stirring before vacuum filtration has been found to give good reproducibility; therefore, it is believed that the reaching of the levelling-off point is the critical issue for the low strength of the enzymatically treated CNF. Indeed, it has been shown in previous investigations that enzymatically treated pulps from the same dissolving pulp, as in Table 2, can reach a tensile index of 169 Nm/g [30,31] and that different CNF materials from bleached kraft pulps can reach a tensile index of 180 Nm/g [32], albeit the fact that somewhat different protocols were used, but that the enzymatic treatment was identical in all references cited here for enzymatically treated nanocellulose samples.

There is some corresponding relationship between the nano-yield and the tensile index when Figure 3 and Figure 4 are compared, but it is certainly not a direct linear relationship between the nano-yield and the tensile strength.

In terms of fibrillation, the carboxymethylated nanocellulose at a high apparent nanocellulose yield has a high content of elementary fibrils with a size of 2.4 nm [33] and the enzymatically treated fibres with a defibrillation energy of 2500 Kwh/tonne is probably fairly similar to what is classified as microfibrillar cellulose (with no charging) and also close to what is conceived as papermaking fines, judging from the results by Fischer et al. [34].

In conclusion, there seems to be a maximum tensile strength for the various CNF materials, peaking approximately around 165–175 Nm/g, close to the extrapolated value of 172 Nm/g at a BET-area of zero m^2^/g. This also suggests, not unexpectedly, that both the BET-area of CNF materials and the film density will be excellent indicators for the state of delamination of CNF-films.

Finally, as briefly indicted above, it is not a surprise that the short span strength of paper made from the same pulp that the CNF-film was made from has about the same strength as the maximum strength of the CNF-film, provided the fibril angle is low.

## 4. Conclusions

This mini-review of the tensile strength of six markedly different nanocellulose materials show that during various extents of delamination tend to approach the maximum tensile strength, proposed from BET-graphs vs tensile index of paper materials based on the same pulp material as the nanocellulose. This review shows that the suggested procedure to estimate the maximum tensile strength is a viable proposition, albeit not scientifically proven. Secondly, there is a relationship between the nanocellulose yield and tensile strength properties, although there may not be a linear relationship between the two measures.

It is also suggested that the determination of the BET-area of nanocellulosic materials should be monitored together with the density of the nanocellulosic materials at different extents of delamination of fibres. It is also stressed that the protocols when comparing mechanical properties between different nanocellulose film materials are widely different in the community, and that progress necessitates uniformity in the laboratory procedures.

## Figures and Tables

**Table 1:** Range of various protocols for CNF tensile strength testing.

Crosshead Speed	1–30 mm/min
Sample width	2–15 mm
Span length	10–50 mm
Basis weights	5–60 g/m^2^

**Table 2:** The different nanocellulose materials used, delamination conc., tensile index at a certain delamination conc., and the original refences, from which the data have been extracted.

CNF-Type	System	Delamination Conc. (≈)	≈Energy (kWh/tonne)	Tensile Strength Index (kNm/kg)	References
**Carboxy methylated** **(D.S. = 0.1)**	1 × 1700 bar	2% (*w/w*)	2400	166 ± 16	[22]
**CMC-grafted** **(D.S. = 0.03)**	5 × 1700 bar	2% (*w/w*)	14,000	150 ± 11	[22]
**CMC-grafted** **(D.S = 0.03)**	5 × 1700 bar	2% (*w/w*)	14,000	164 ± 12	[23]
**Enzymatic** **(D.S. = 0.005)**	5 × 1700 bar	2% (*w/w*)	12,400	107 ± 6	[22]
**Phosphorylated** **D.S. = 0.17)**	3 × 1700 bar	2% (*w/w*)	7200	143 ± 12	[24]
**Sulfoethylated** **(D.S. = 0.11)**	1 × 1700 bar	2% (*w/w*)	2400	138 ± 5	[25]
**Alkoxylated** **(D.S. = 0.11)**	1 × 1700 bar	2% (*w/w*)	2400	170 ± 9	[26]
